# Migrant mothers, left-behind fathers: the negotiation of gender subjectivities in Indonesia and the Philippines

**DOI:** 10.1080/0966369X.2016.1249349

**Published:** 2016-10-27

**Authors:** Theodora Lam, Brenda S. A. Yeoh

**Affiliations:** a Asia Research Institute, National University of Singapore, Singapore; b Department of Geography, National University of Singapore, Singapore

**Keywords:** Left-behind father, left-behind children, changing gender roles, web of care, feminized labour migration, Padres dejados atrás, hijxs dejadxs atrás, roles de género cambiantes, red de cuidado, migración laboral feminizada, 留在家乡的父亲, 留在家乡的儿童, 改变中的性别角色, 照护网络, 女性化的劳动迁徙

## Abstract

The distinct feminization of labour migration in Southeast Asia – particularly in the migration of breadwinning mothers as domestic and care workers in gender-segmented global labour markets – has altered care arrangements, gender roles and practices, as well as family relationships within the household significantly. Such changes were experienced by both the migrating women and other left-behind members of the family, particularly ‘substitute’ carers such as left-behind husbands. During the women’s absence from the home, householding strategies have to be reformulated when migrant women-as-mothers rewrite their roles (but often not their identities) through labour migration as productive workers who contribute to the well-being of their children via financial remittances and ‘long-distance mothering’, while left-behind fathers and/or other family members step up to assume some of the tasks vacated by the mother. Using both quantitative and qualitative interview material with returned migrants and left-behind household members in source communities in Indonesia and the Philippines experiencing considerable pressures from labour migration, this article explores how carework is redistributed in the migrant mother’s absence, and the ensuing implications on the gender roles of remaining family members, specifically left-behind fathers. It further examines how affected members of the household negotiate and respond to any changing gender ideologies brought about by the mother’s migration over time.

## Resumen

La distintiva feminización de la migración laboral en el sureste de Asia –en particular en la migración de madres que ganan un ingreso con el trabajo doméstico y el cuidado en mercados laborales globales segmentados por género– ha alterado significativamente los acuerdos del cuidado, los roles y prácticas de género, así como las relaciones intrafamiliares dentro del hogar. Tales cambios se experimentan tanto para las mujeres migrantes como para los miembros de la familia dejados atrás, particularmente quienes son lxs cuidadorxs ‘sustitutxs’, como los esposos que se quedan. Durante la ausencia de las mujeres en el hogar, las estrategias de mantenimiento del mismo tienen que ser reformuladas cuando las mujeres-como-madres reescriben sus roles (pero a menudo no sus identidades) a través de la migración laboral como trabajadoras productivas que contribuyen al bienestar de sus hijxs vía el envío de remesas y la ‘maternidad a larga distancia’, mientras que los padres y/u otros familiares a cargo que se quedaron deben asumir las tareas dejadas por las madre. Utilizando material de entrevistas tanto cuantitativas como cualitativas con migrantes que regresaron y miembros del hogar que se quedaron en las comunidades de origen en Indonesia y las Filipinas, que experimentan presiones considerables de migración laboral, este artículo explora cómo el trabajo del cuidado se redistribuye en ausencia de la madre migrante, y las consiguientes implicancias sobre los roles de género de los miembros de la familia restantes, específicamente sobre los padres que se quedan. También estudia cómo los miembros afectados del hogar negocian y responden a cualquier cambio en las ideologías de género causado por la migración de la madre con el tiempo.

## 摘要

东南亚劳动迁徙的显着女性化——特别是负担家计的母亲在性别划分的全球劳动市场中，迁徙他乡从事居家和照护工作——已大幅改变了家户中的照护安排、性别角色与实践及家庭关係。迁徙的女性以及留在家乡的家庭成员，皆经历了这些变迁，特别是诸如留在家中作为’替代’照护者的丈夫。当这些女性在家中缺席时，作为母亲的移工女性透过劳动迁徙成为具生产力的工人，并以经济汇款和‘远距母职’对儿女的福祉做出贡献来重新书写她们的角色（但却经常不是她们的身份认同），而留在家乡的父亲和／或其他家庭成员需进一步承担母亲离开后的部分任务时，持家的策略必须重新形塑。本文运用在经历劳动迁徙的巨大压力的印尼与菲律宾的劳动力供给社群中， 对回乡移民与留在家乡的家户成员所进行的量化与质化访谈材料，探讨照护工作如何在移工母亲缺席时重新分配，及其对留下的家庭成员——特别是留在家乡的父亲——的性别角色之意涵。本文进一步检视家户中受到影响的成员，如何随着时间协商并回应母亲移居他乡工作所带来的任何性别意识形态的改变。

## Introduction

Transnational labour migration is a livelihood strategy used by many Southeast Asian households, especially in Indonesia and the Philippines since the 1980s and 1970s respectively, to improve their family circumstances (Silvey 2007a; Battistella and Asis 2013). The high numbers of female migrants (over 50% per country, see Graham and Yeoh 2013) – many of whom are mothers – and their consequent long absences from the home have since led to heightened anxieties over the potential ‘care deficit’ in the home as well as a complex redefinition of economic and gender roles for both men and women in larger society and within the family realm. On the one hand, the so-called feminization of labour migration in these Southeast Asian countries may potentially destabilize the traditional power balance between genders, leading to a renegotiation of the patriarchal bargain and a more egalitarian gender division of household labour and carework in sending communities (even as the ready supply of migrant women as domestic and care workers rigidify the gender divide in household labour in receiving societies). On the other hand, the extent of social transformation may be limited by the persistence of gendered ideologies pertaining to parental identities and roles even when migrant mothers and left-behind fathers swap roles and modify their traditional caregiving practices.

Much has been written on women from less developed countries migrating to plug the caregiving gaps in richer economies within a global chain of care labour. More recently, attention has also shifted to the web of care surrounding children left behind by migrant mothers. However, literature on caregiving and the changing gender roles of left-behind family members, particularly men, in the women’s absence remain fairly sparse. In this vein, the article investigates the (re)distribution of carework when women respond to the economic pull of new feminized migrant labour streams by first identifying the children’s main/primary ‘surrogate’ carer. It then probes into the transformations of gender roles and subjectivities, specifically that of left-behind fathers’, in the wake of the mother’s migration and their responses to the changing gender practices in their families. The spotlight is placed on men’s role in carework and social reproduction, and how changing gender roles affect the ideologies and practices of fatherhood and masculinities. Responding to Pessar’s (2005, 4) call for ‘more research on the ways in which stay-at-home husbands and fathers adjust their masculine identities, practices, and domestic power dynamics to conform to’ their altered situations when wives and mothers migrate, this article thus explores the impact of women’s migration on left-behind men’s negotiation of gender roles, subjectivities and relations in dealing with the care of their children. Finally, it questions if the transformations in the gender roles and subjectivities of both migrant women and left-behind members due to women’s migration are sustainable.

## Changing care arrangements and gender subjectivities in the wake of migration

Care encompasses the work of looking after one’s ‘physical, psychological, emotional and developmental needs’ (Standing 2001, 17). Embracing ‘a range of human experiences and relationships of obligation, trust, loyalty and commitment concerned with the well-being of others’, it can be differentiated by ‘quality, quantity, type of caregiver, care receiver and so on’ (Kofman and Raghuram 2009, 3). Often regarded as invisible and undervalued, care(work) has become increasingly commodified and divided in recent years (Glenn 2000; Yeoh and Huang, forthcoming). This is evident as working women in more developed economies try to plug the care deficits in their own homes often by outsourcing physical aspects of care to less privileged women from poorer economies in the South whilst personally retaining the emotional/nurturing aspect of care in what Hochschild (2000) terms as a ‘global care chain’ (GCC).

While the absence of any one parent from the household is likely to cause some ‘displacement, disruptions and changes in caregiving arrangements within the family’ (ECMI/AOS-Manila, SMC, and OWWA 2004, 61), the migration of mothers from the Southern, less developed end of the GCC, has been noted to require greater adjustments in caregiving roles as compared to the father’s absence. This is largely due to the gendered division of labour where women such as those in Indonesia and Philippines are still traditionally regarded as the main nurturers responsible for the upkeep of their households as well as physical and social reproduction in their communities (Geertz 1961; Zlotnik 1995; Sen 1998; Medina 2001). The numerous negative reports of left-behind families in the mass media also suggest that families often suffer a greater sense of loss when mothers go overseas to work. Nevertheless, some scholars have argued that claims of the alleged ‘care crisis’ may have been exaggerated as the detrimental impacts of transnational migration on the emotional and social aspects of family relations are not predetermined (Graham et al. 2012).

When fathers migrate with the aim of fulfilling and continuing their designated breadwinning care role (Oetomo 2000; Osteria 2011), the family’s caregiving arrangement is found to remain fairly stable with mothers continuing in their roles as caregivers. Left-behind Filipino women, for example, juggle the tasks of taking over their migrant husbands’ roles, caring for their children and successfully preserving their existing nuclear household structure (Battistella and Conaco 1998; ECMI/AOS-Manila, SMC, and OWWA 2004; Parreñas 2005). Though left-behind women found themselves taking on a wider range of roles and responsibilities – often translated into heavier workloads and additional stresses or hardships – they may also become more autonomous and involved in decision-making within the family, gaining greater self-confidence in the process (see Dwiyanto and Keban 1997; Battistella and Conaco 1998; Skeldon 2003).

Concurrently, migrant men, such as Filipino seafarers in McKay’s (2011, 3) study, appeared to have capitalized on their migration and occupation status to ‘transgress certain gender roles or appropriate competing gendered practices but without serious stigma or challenge to their overall masculinity’. Drawing on Townsend’s (2002) concept of the ‘package deal’,^1^ McKay (2011, 4) argued that Filipino seafarers embody the ‘Filipino package deal’ of successful manhood as their peripatetic jobs allow them to fulfil both the economic and non-economic elements of masculinity such as ‘work, breadwinning, family and community respect’ as well as ‘risk-taking, physical aggressiveness and worldliness’. He further proposed that Filipino seafarers constructed a ‘hegemonic masculine privilege’ grounded on providership which gave them the chance, proficiency and flexibility to cross gender divides and appropriate other gender practices without repercussions.

Returning to the impact of female migration on care arrangements and gender subjectivities, existing studies revealed a more diversified model of care when mothers migrate. There is still considerable debate about the extent and sustainability of the involvement of non-migrant fathers in caregiving, especially after mothers return. While some earlier studies have portrayed left-behind fathers in Asia as shunning nurturing roles and engaging in drinking and drug-taking habits as a form of escape from their reversed emasculated situation (see Gamburd 2005; Parreñas 2005), more recent work has shown fathers playing an increasingly important and competent role as left-behind carers (Hoang and Yeoh 2011; Lam and Yeoh 2014). Indeed, the literature reveals that left-behind Bangladeshi, Indonesian, Filipino, Sri Lankan and Vietnamese fathers do assume ‘mothering’ roles to varying degrees, and this may be an increasing trend in some parts of Asia where nuclear families are becoming more prevalent. Left-behind men also appear to take on caregiving and childcare roles with the assistance of other family members such as older children and/or other female relatives (Chantavich 2001; Afsar 2005; Hugo 2005; Parreñas 2005). The observation that there was actually more Sri Lankan male participation in the household and child-rearing tasks than reported prompted Gamburd (2000) to postulate that older concepts of gender roles may in fact be slowly changing. This sentiment is also echoed in Pingol’s (2001) study of Filipino migrant wives and househusbands which recounted how fathers may, in their own way, become important care providers for the household and children.

Changing care arrangements in source communities with men becoming increasingly visible in caregiving roles raises questions as to whether men are redesigning their masculine selves. Pingol (2001) was among the first to argue that assuming care duties constituted a way for left-behind men to reclaim, as well as to reinvent, their masculinities. McKay and Lucero-Prisno III (2012, 23) further noted that it was by ‘appealing to broader masculine ideologies of “being in control” and “maintaining autonomy”’ that left-behind fathers – stripped of their breadwinning responsibilities – conserve a semblance of their role and identity as heads of households. Drawing on McKay’s (2011) formulation of ‘package deal’ masculinity, we work through the way the ‘left-behind father package’ is negotiated around childcare arrangements in Southeast Asia where the increased demand for feminized migrant labour has led to the appropriation of family providership roles by migrant mothers.

## CHAMPSEA study and methodology

This article utilizes both quantitative and qualitative data from a study entitled ‘Child Health and Migrant Parents in South-East Asia (CHAMPSEA)’, a mixed-method study investigating the impacts of parental migration on child’s health and well-being in Southeast Asia.^2^ The quantitative data is derived from surveys conducted in 2008 with 1034 Indonesian (East and West Java) and 1000 Filipino (Laguna and Bulacan) responsible adults (RA), carers (who may also be the RA) and older index children (IC). Respondents come from transnational and non-migrant households of roughly equal proportions, with at least one IC in one of two age groups: 3–5 year-olds (preschool or young children)^3^ and 9–11 year-olds (primary school aged or older children). To ensure a more focused analysis of the data given resource constraints, CHAMPSEA only sampled ‘intact, heterosexual families’, and the exclusion of single-parent or other family types may be its greatest limitation.

The neighbouring countries of Indonesia and Philippines were chosen as they share various commonalities in cultural traditions and lifestyles. Importantly, both countries have long migration histories characterized by high rates of female transnational labour migration, generating a major supply of domestic and care workers for the more developed economies in Asia and the Middle East (Asis 2006; Silvey 2007b, 2009; Battistella and Asis 2013). While there are cultural and religious differences between the predominantly Christian Philippines and the largely Muslim Indonesia, there are also major similarities when it comes to family life. In particular, by inscribing disparate roles for women and men, both religions reinforce gendered discourses on the family and thus perpetuate, rather than challenge, traditional breadwinner models manifested in public representations. As Velayutham and Wise (2005) argue in their study of migrants from a South Indian village, transnational migration creates a moral economy predicated on discursively constructed ideals of the family, which may operate to reproduce ‘tradition’. This is reflected in the ways in which caring roles are imagined both in the public domain and in the private spaces of the transnational family. It is also observed in both countries that the goal of securing the family’s economic well-being through the migration of one or more members has taken root as a commonly accepted strategy, sometimes taking precedence over traditional family and gender ideologies. Such similar motivations and dynamics of migration may have the effect of flattening out stark differences between the two countries in terms of ‘doing’ family.

In the follow-up to the survey, interviews with 52 Indonesian (from Tulungagung and Ponorogo) and 48 Filipino carers (from Laguna), as well as semi-structured interviews with 32 older children (16 from each country) were conducted in 2009. Additional interviews with key members, namely returned-migrants, left-behind carers and older children, from 20 returned-migrant households were conducted between 2009 and 2012. Overall, 22 Indonesian and 13 Filipino left-behind father-carers^4^ were among those interviewed. Interviews were mainly conducted in native languages and translated into English. Table 1 presents the sample of mother-migrant households surveyed or interviewed.

**Table 1.** Indonesian and Filipino mother-migrant households surveyed and interviewed.

	Indonesia	Philippines^a^
Quantitative study	*N*	*N*
Mother-migrant households with young IC (3–5 year-olds)	143	28
Mother-migrant households with older IC (9–11 year-olds)	150	66
Qualitative study (from mother-migrant households only)		
Left-behind carer	28	23
Returned-mother-migrant	8	6
Older IC (9–11 year-olds)	15	15

a The difficulty of recruiting married, female-migrants with children matching CHAMPSEA’s sampling criteria from the Philippines’ selected field sites was only uncovered midway through the fieldwork.

## Care arrangements in Indonesia and the Philippines when mothers are away

As discussed elsewhere, the project found mixed care arrangements for left-behind children when one or both parents migrated (Hoang et al. 2015). The prevalence of traditional gender norms in assigning nurturing roles to women and breadwinning roles to men is apparent: mothers continued to be the main carers of children when both parents lived at home^5^ (90.0%) or when fathers were away (93.6%). Focusing specifically on the Indonesian and Filipino sample, mothers were the key persons responsible for the daily care of the large majority of children living in non-migrant households (90.6% in Indonesia and 92.4% in the Philippines). Mothers were also the principal carers when fathers migrated overseas to work (96.5% in Indonesia and 91.5% in the Philippines).

When mothers assumed the roles of the overseas breadwinner, the care arrangements for the children featured a more visible proportion of non-parental carers although the majority were cared for by their fathers (67.9% in Indonesia and 59.6% in the Philippines). The relatively high number of left-behind fathers being reported as the main carer in the majority of mother-migrant households in CHAMPSEA is one distinctive finding of this study.^6^ As described by Hoang and Yeoh (2011) and Lam and Yeoh (2014), more men were stepping forward to assume mothering roles during their wives’ absence and were proactively involved in childcare. Nonetheless, Indonesian and Filipino households also enlisted the help of other non-parental family members such as maternal and/or paternal grandparents, aunts and uncles, other relatives, left-behind children’s older siblings, and to a minimal extent, non-family domestic workers in providing care when the mothers are away. Among the sample, there is still a preference for female non-parental carers over male counterparts as females constitute the larger proportion of non-parental carers. While the presence of these non-parental carers confirm the importance of ‘other mothers’, the preference for female-carers also reflect the persistence of gendered thinking in care negotiations for the children in these countries.

## Repacking the ‘package deal’: gender subjectivities of the left-behind father

### The responsible father

While men’s role as breadwinners and providers – key elements of the original ‘package deal’ – is deeply ingrained into contemporary Southeast Asian societies, it is being increasingly challenged by the growing female participation in the labour force. The masculine identities of Indonesian and Filipino fathers predicated on providership are apparently shifting with the migration of women, often mothers, leaving the men (or other family members) to care for the household and children. Left-behind fathers are thus not only confronted with the loss of their breadwinning status, but also with the added burden of having to take over the caregiving roles vacated by their migrant wives. Under these circumstances, left-behind men in this study began to rationalize the ‘package deal’ and rework caregiving as a form of redemption to lessen their personal feelings of inadequacy and embarrassment at their inability to perform the expected male breadwinning role. As Raul^7^ (50,^8^ Filipino, RMMFC^9^) puts it,

It’s [the care arrangement] really hard but what can we do? … I don’t really have a hard time [in the actual care of my children] but what I feel bad about is my failure to assume my responsibility. I should be the one working.

Although these Filipino father-carers were unable to escape the traditional viewpoint articulated in the phrase *haligi ng tahanan* (literally translated as ‘pillar of the home’) that referenced fathers as providers, and while caring for children was not considered a ‘real’ job, they felt that they should at least redeem themselves (‘assume my responsibility’) by contributing to the family through caregiving.

Despite the general feeling of having ‘no other choice’ but to assume the role as the children’s main carers when mothers were away since ‘nobody else can help me’ care for the family, left-behind fathers were cognizant that by agreeing to their wives working overseas, they were partly responsible for their current situation. Given their failure to retain the main breadwinner status as a result of gender segmentation in the global demand for domestic workers and care-workers, the fathers were aware of their own ‘low’ or ‘mismatched’ qualifications vis-à-vis global labour market demands (‘her salary [as a domestic worker] would be higher’, or ‘her visa was approved first’). Nevertheless, many Indonesian and Filipino father-carers were quick to stress their masculine qualities as reliable and responsible fathers over their diminished breadwinning capability. Matius (45, Indonesian, RMMFC), for example, said, ‘I felt myself as the only parent at that time to look after children, but I did it as usual, composedly, because I’m very responsible for my duty’.

Even when alternative help from the grandmother was available, Erwin (39, Filipino, RMMFC) decided with his wife that he would be the main carer of his children because ‘I [Erwin] am also here’ and ‘there could be no other one to look after the children but me’. Udin (32, Indonesian, MMFC) finally resolved the conflicting moral struggle and dilemma over the household division of providing monetary care through breadwinning vs. intimate care by balancing constructions of ‘responsible’ masculinity with community/cultural expectations and religious/moral instructions on familial duty:

… it was planned that both of us would go to Arab Saudi. Husband and wife were possible to be in the same place, right? At Arab Saudi, I would drive and she would do domestic job. But, we thought about it again, about the love the children received would be less than enough … if they would be entrusted to only their grandmother. Then, I solved it! If it was both of us going to Arab Saudi, who would take care of the children, who would educate them? It would be less than enough. But if it was only one of us going to Arab Saudi, it [salary] wouldn’t be enough. Then, I went to [the *imam* or religious leader] to have a consultation. It was better [for my wife] to go to Taiwan, the salary was big there. … But I did what is dictated by Allah.

Here, fathers-as-men clearly exerted their agency through the malleable language of ‘responsibility’ and tried to unify the ideals and practices of being a ‘good’ father with those of being a ‘good’ man. Conserving their masculine selves hence involved simultaneously compensating for their deficiency in the providership care element of the ‘package deal’ by living up to other moral expectations tied to Asian familism as responsible fathers.

### The productive man

Left-behind fathers also believed that they could not afford to rest on their laurels when their wives were working overseas, and that both parents should collaborate in contributing to the financial well-being of the family regardless of the relative size of their contributions. This is evident when the majority of father-carers (83.3% in Indonesia and 64.3% in the Philippines), in the absence of their breadwinning wives, reported themselves as ‘working’ even though they were their children’s primary carers.^10^ This is consistent with Hoang and Yeoh’s (2011) finding that while Vietnamese left-behind fathers did not shun care-work, the large majority persisted in balancing childcare with some form of paid work in order to preserve their masculinity and pride in the face of their migrant wives’ increased economic power. They concluded that paid work in the context of left-behind men ‘serves to ward off potential ridicule arising from men’s engagement in “women’s work,” counteracting any demasculinization effects that this new arrangement may bring’ (Hoang and Yeoh 2011, 733). This argument also finds some reverse congruence with Gamburd’s (2000) observation where left-behind Sri Lankan husbands without regular jobs tended to feel a strong sense of inadequacy, leading them to indulge in ‘vices’ such as drinking, gambling and womanizing. Veronica (34, Filipino, RMMFC) confirmed the importance of work for her left-behind husband regardless of the low wages, ‘He feels embarrassed for my parents to say that just because his wife is in Bahrain, he would not work. So even if it is hard, he cannot stay at home’.

Being economically productive even when the returns did not add significantly to the household income also had the advantage of helping Indonesian and Filipino left-behind fathers cope with the ‘stress’ of having time on their hands, as well as earned them respect and standing in the community to partly compensate for their loss of status as left-behind spouses. Sukmo (42, Indonesian, RMMFC) worked in the field while Raymond (40, Filipino, RMMFC) drove a tricycle and worked for the barangay as well as the local church to ‘lessen stress’ and avoid thinking of their ‘problems’ or ‘wife’. Overall, masculine subjectivities remained indivisible from the social politics of fatherhood as ‘men’s authority in the family’ and their breadwinning function are positioned as the crux of masculinity politics (Hobson and Morgan 2002, 5). Remaining productive, even as productivity constitutes a form of care, continues to constitute the cornerstone of masculine ideals.

### The capable ‘mothering’ father

Besides engaging in outward-oriented care such as buying food, managing finances, earning money, attending school events, and administering discipline, left-behind fathers professed that they were also involved in the intimate and mundane aspects of carework in relation to their children. Many left-behind Indonesian fathers were prepared to *be* mothers by cooking, feeding, doing the laundry, bathing and supporting children in their studies (Hoang, Yeoh, and Wattie 2012). Similarly, as an illustration of what Filipino left-behind father-carers took on as carework, Eric (38, Filipino, MMFC) shared:

With regard to doing assignments and projects, I’m the one [who helps the IC]. For a project, or for example, as long as I’ve fixed [the other children] and fed them, bathed them, before sleeping. When they’re already in bed, that’s the only time I help him with his project. If he can’t do it anymore, I let him sleep and I finish his project. I stay up until four in the morning. … Whatever his mother used to do, that’s what I do. I prepare the breakfast; I cook for all of us. I do everything … I’m the one who takes them to school. After I take them to school, I drive for hire for two hours. Then I go home and cook. After cooking, I go back to the school. If I still have time to drive for hire, I do so. Then I fetch them. … Even if for example, I’m feeling cold due to fever … even when I feel bad, I force myself to get up to be able to take care of their needs.

When questioned if fathers needed to be taught how to carry out these mundane aspects of care which were seen to come ‘naturally’ for mothers, both return mother-migrants and father-carers were quick to affirm the father-carers’ capabilities in these areas. While some men may have picked up housekeeping skills from their own migration journeys, the long-standing trend of female labour migration in these two countries may in fact have prepared the men for the inevitability of this prospect since they were young. Sari (35, Indonesian, RMMFC) emphasized,

Yes, they know. Every man in my *kampung* (village) of course must know [how to cook and look after children]. So many women [migrate]. Yes! Every house in my place is all the men are alone. Maybe because the women aren’t there. Maybe from young, the mother already teach [them]. The husbands do everything in the house, the cooking, the cleaning, the husband will do everything.

Father-carer, Uji (25, Indonesian, MMFC) confirmed, ‘it was usual for me to wash the laundry … [and] when my wife was busy, I would cook’.

Overall, the majority were able to say confidently that while they may lack the ‘woman’s touch’ in cleaning and caring, they were still able to provide moral, emotional and physical care, and be both ‘father’ and ‘mother’ to their children. Sadewa (50, Indonesian) a return father-migrant who was also formerly a left-behind father-carer with a migrant wife, declared, ‘I can do male and female jobs. I can cook, I can wash. No problem’. Mothers and children were also quick to affirm father-carers’ good works: ‘He [father-carer] is doing all the work that I was supposed to do. He fulfilled those roles especially to my little boy, he has become both the father *and* the mother to him’ (Veronica); ‘[I would still choose *ibu* (mother) to be the migrant] because father can take care of both the house and children’ (Yuda, 12, Indonesian, RMMFC).

Within the span of a decade, the accounts in this study are revealing a rather different portrait of the left-behind father as compared to the unemployed and distant left-behind men described in Parreñas (2005) study conducted from 2000 to 2002 in central Philippines. While the results may be simply different, the evidence from this study suggests a gradual change in fathering practices over time given the intensification of female labour migration. Increasingly, left-behind fathers are beginning to take pride in their versatility and capability in taking on board the day-to-day, mundane aspects of social reproductive work associated with ‘mothering’ while retaining their identities as fathers. While a product of the force of circumstances, the ‘mothering’ father is no longer a social oddity but a somewhat heroic role which both left-behind men and migrant women jointly conspire to uphold in order to further transnational migration projects which secure the economic wherewithal of the family.

### The man is in control!

Pingol (2001) has argued that the key for Filipino men with migrant wives to upholding their masculine identity was to retain ‘control’. In this study, this control was not only manifested in their choices, the roles they assumed and their performance of these roles; it was also evident in the way men do gender, presented themselves and explained their circumstances. For example, when it came to using remittances, many father-carers claimed that they themselves decided how to use the money and that the money was mainly meant for their children. Mario (43, Filipino, MMFC) insisted, ‘Because I’m not like other husbands who would ask money for themselves. With me, I’m used to hardships. What I only ask for, for my children, electricity, and water’. Even in cases where the evidence from other sources pointed to the fact that it was their wives who decided on migrating, left-behind fathers invariably would assert their power and control over family decisions by placing the emphasis in their narratives on ‘I’. The rhetoric as in ‘*I* also wanted her to go, to earn a little more than here. It will also help my children’; ‘*Me, myself* decided [not to rely on a helper]’; ‘*I* want to take care of the children’; ‘*I* can do it by myself. *I* can do household chores’; and ‘*I* can get money.’ is used to affirm men’s masculinities and control over their lives, wives and decisions. The ability to retain and manifest control remains an important part of the ‘package deal’.

Indonesian mother-migrants were also quick to clarify that while they initiated or decided on the idea of migration, they only migrated with their husband’s permission.

I thought about that [migration] alone. If I do not have money, how would I support my children’s life? And then I communicated it to my husband, I asked him whether or not I would be allowed to work overseas. We talked about that, he wanted to go also. But if he did not allow me, I would not have worked overseas. … The important thing is my husband, I left because he allowed me. If he did not allow me, I would not have worked overseas. (Harum, 34, Indonesian, RMMFC)

Sari concurred,

Because I’m married, my husband is number one … At least my husband is good, I must respect the husband. Husband is the provider. After that, he said, ‘it’s okay. Because this is for our food, for our son’s future’. Maybe he thinking ok, this is quite good so let me go.

Women subtly ‘bargained’ for their departure from home and abdication of traditional caring duties by according men the seat of control in the household. They were complicit in ensuring that a modified form of domestic patriarchy continue to prevail by protecting men’s image of their own masculinities such that their husbands – whom they had to depend on to shore up the domestic front in their absence – would not feel threatened when relinquishing the breadwinner’s position to their wives.

## A fragile and (un)sustainable package

Lest the image of a left-behind father begins to take on superhero proportions, not all is well in the left-behind household, especially when fathers are unable to rely on other kin for help. Whilst father-carers were quick to reassure their migrant spouses about their children’s well-being, several men felt ‘weakened’ by the ordeal and reported experiencing stress and even health problems during their wives’ absence. Bakti (36, Indonesian, RMMFC) confided,

I had health problems lately. One time, I just stayed at home without working for about four and half years … when she was working overseas. When my wife was away, I tried to find treatment for myself. I went to an alternative healer, a doctor, a religious alternative healer, but it might be caused by myself that I was exhausted, and I had a problem also with my friend which became a burden for me to think about. I told her [my wife about my problems], but not all information like I stayed in the hospital twice. I was also afraid that I could make her think or worry about me and she could fall sick also.

Bakti’s story resonates with quantitative findings from the study (using the SRQ composite scores) which noted ‘that the existence of a probable psychological vulnerability is higher among Indonesian father-carers (30.8%) than mother-carers (26.9%) when their spouses are away’ (Lam et al. 2013, 426).

Some father-carers also confessed (as affirmed by their children’s accounts) to turning to aberrant activities such as gambling and drinking to ease their loneliness and stress. Father-carer Augustin (37, Filipino, RMMFC) admitted sheepishly to overspending his wife’s remittances on ‘happy times’ such as gambling and drinking during his wife’s absence, causing her to call and ‘shout’ at him. Duly reprimanded, Augustin then promised he would try to change: ‘It is not actually difficult. I just think of my children growing up and when I discipline them, they wouldn’t obey me because I’m not a good example’. Unfortunately, a chain of disastrous events chiselled away at Augustin’s resolve to affirm his fatherhood by being ‘a good example’: their oldest daughter ran away from home and became pregnant when the mother had just left for overseas, their middle child – a son – also dropped out of high school after missing too many classes due to stomach pains (later discovered by his returned-mother-migrant to be a result of a urinary tract infection), and their youngest child with special needs – another daughter – had to stop school as there were no adults available to send her there. While the mother-migrant had to return home to take care of these problems, it was instructive that she did not hold Augustin responsible for the misfortunes. Instead, she reasoned that her eldest daughter was simply ‘hard-headed’, that her son had – for unknown reasons – not told his father about his ailment, and that the special school for her youngest child was really too far. Mother-migrants were hence complicit in making excuses for problems that occurred during their absence, as they were dependent on left-behind carers soldiering on with care duties to preserve the opportunities for them to work abroad.

The demands and type of caregiving needed from the fathers are also changing as children, particularly daughters, mature and undergo transformations brought about by puberty and adolescence. Most father-carers interviewed, as men, felt uncomfortable in caring for their teenage daughters especially when they began menstruating. In these cases, they expressed a strong preference for mothers to be present. Matius observed, ‘As her father, I think I’m not in the right position to teach her’. Puberty was a rite of passage that could turn father-daughter relationships awkward as men exhibited fear and great discomfort dealing with such issues. Dante (51, Filipino, MMFC) further grumbled about the woes of a man caring for a daughter,

… her mother should be the one in school. I’m the one who almost does everything … from the things she has to wear, and of course she’s female! Never mind if my child is a male, I can handle that. But I got used to it eventually. At the start, it’s really like that … I ask my female friends, ‘What should my daughter wear? You’re female, teach me!’

Raul considered the different challenges of caregiving as his daughter grew older,

this girl needed more caring when she was younger but not anymore. Now that they’re all grown up, they can do many things by themselves. There is no problem with this girl when she’s at home; the problem comes when she goes out somewhere.

Others lamented the need to spend longer hours at home, thereby sacrificing leisure activities and the freedom to spend an evening out bonding or relaxing with friends. Sukmo, for example, complained, ‘It’s hard to be a mother because I’ve to do everything: cooking … going to the field … go home without rest and then cook and sometimes, washing’. Some fathers laughingly revealed that needing to spend more time on childcare at home actually forced them to give up or reduce their drinking activities. Romy (43, Filipino, MMFC) declared, ‘before, I used to have social drinking, but now sometimes I couldn’t do it especially during the night. If I do that, what will happen to my kids?’

Despite the positive attitude father-carers in the study held for their caring duties, they also expressed considerable relief in having their wives home: ‘[When my wife comes back] … my burden is lessened. … I don’t have to cook before I work, so when I work I don’t need to think about cooking … have no burden. … I’ve more free time’ (Soleh, 38, Indonesian, RMMFC). Said Matius, ‘Yes, I’m free now to do what I want to do, but only positive things. I’ve about 50% less burden unlike before’. Pablo (45, Filipino, RMMFC) was ‘very, very’ happy when his wife returned and acknowledged the importance of a ‘woman’s touch’ in the household,

The way a woman cleans the house or takes care of the household is quite different from the way a man does it. The mother’s way is quite different … There are many changes [in IC]. When I used to wake her up to prepare for school, I needed to wake her up for about ten times before she got up. But now when her mom wakes her up, the mom just has to say, ‘child, wake up’ and [she] gets up [at once].

Ismael (30, Filipino, MMFC), another father-carer, would gladly return the reins of caregiving to his wife as he felt that ‘a mother’s care is really different from mine because they’re more knowledgeable. The way we take care of him is really different. Maybe, she’ll double what she’s supposed to do … She’s really focused [on the children]’.

## Conclusion: gendered nature of care

As Kofman and Raghuram (2009, 18) have noted, the division of care labour in society ‘refracts and reproduces existing social hierarchies’. At the southernmost end of the care chain and in the wake of prolonged feminization of labour migration, childcare practices have evolved to draw in left-behind father-carers in the absence of mothers. Stripped of their main provider status, left-behind fathers are compelled to create a new ‘package deal’ to reconstruct their masculine selves by building on other characteristics such as responsibility, adaptability, capability and control. As we have shown, the redefined ‘package deal’ centres on retaining a sense of power and control over their changed circumstances. In their new roles as carers, fathers expressed a sense of pride when they could claim that their children were doing well under their charge. While ‘good fatherhood’ is strongly anchored on the ability to be successful providers of material welfare, their strengths in ‘overcoming the odds’ in ensuring that their children were thriving under their watch featured prominently in their construction of masculine selves. At the same time, while accomplishing their caring duties well is considered a key component of the new ‘package deal’, being engaged in some form of productive work outside the home continues to be significant in protecting their self-image as men who are actively and substantially contributing to their families’ welfare. Holding on to income-generating work and having a web of other carers to depend on in looking after their children are hence important dimensions of fathers’ ability to provide sustained care for their children. The expectation that role-reversal is probably ‘temporary’ also helps them retain a sense of control. As Pingol (2001) and Añonuevo and Estospace (2002) note, left-behind men do not lose their masculinities but seek to reclaim them in other ways. In the process, they create alternatively packaged versions of what constitutes ‘good fatherhood’ and ‘good man’ to counter hegemonic notions of masculinity that restrict men to breadwinning roles.

While gendered *practices* of care at the end of the care chain have undergone important shifts toward more flexible patterns in the absence of mothers, there is no clear evidence at this point to indicate revolutionary transformation in gender *ideologies* around parenthood and childcare. Left-behind father-carers in the study still prefer migration/work to taking over the mothers’ roles, showing that they are still generally uncomfortable in radically changing existing gender ideologies and household division of labour. While feminized transnational migration has opened up a ‘new space representing newly emergent assemblages of gender, power, economics and cultural ideals that may put pressure on men to perform their masculinities differently, or at least more flexibly’ (Yeoh and Ramdas 2014, 1203), ‘deep-seated transformations in gender ideologies or scripts are … more resistant to change’ (McIlwaine 2010, 281).

Despite major structural shifts wrought by globalization and the speeding up of mobilities in Southeast Asia, the family as a living arrangement underwritten by both the sharing of material resources and ties of intimacy remain relatively resilient if ever-changing. Normative gender practices and identities are in flux as people constantly ‘do’ and ‘un-do’ gender in everyday life. Within this highly mobile world, fathers, like mothers, actively adapt their gender practices and identities in small steps, even as broader ideational change progresses more slowly. In time, as suggested by Peng and Wong (2016, 2031), the new discourse of simultaneously ‘being a mother and a father’ may erode the existing gendered views associated with what is commonly identified as feminized or masculinized tasks. In the era of migration and family survival, ‘doing family’ may thus become more important than ‘doing gender’.

## Notes on contributors

***Theodora Lam*** is a postdoctoral fellow in the Asia Research Institute, National University of Singapore and a writing fellow at the Department of Socio-Cultural Diversity, Max Planck Institute for the Study of Religious and Ethnic Diversity. Her research interests cover transnational migration, children’s geographies and gender studies. She has co-edited several special journal issues on transnational families in *Global Networks* (with Brenda Yeoh and Shirlena Huang) and *International Migration* (with Shirlena Huang and Brenda Yeoh) and published in various journals and edited books including *Children’s Geographies, Environment and Planning A, and Asia Pacific Viewpoint*.

***Brenda S. A. Yeoh*** is Professor (Provost’s Chair), Department of Geography, as well as Dean of the Faculty of Arts and Social Sciences, National University of Singapore. She is also the Research Leader of the Asian Migration Cluster at the Asia Research Institute, NUS. Her research interests include the politics of space in colonial and postcolonial cities, and she also has considerable experience working on a wide range of migration research in Asia, including key themes such as cosmopolitanism and highly skilled talent migration; gender, social reproduction and care migration; migration, national identity and citizenship issues; globalizing universities and international student mobilities; and cultural politics, family dynamics and international marriage migrants. Her latest book titles include *The Cultural Politics of Talent Migration in East Asia* (Routledge, 2012, with S. Huang); *Migration and Diversity in Asian Contexts* (ISEAS press, 2012, with A.E. Lai and F. Collins); *Return: Nationalizing Transnational Mobility in Asia* (Duke University Press, 2013, with B. Xiang and M. Toyota); *Transnational Labour Migration, Remittances and the Changing Family in Asia* (Palgrave Macmillan, 2015, with L.A. Hoang); as well as a paperback reprint of her book, *Contesting Space in Colonial Singapore: Power Relations and the Urban Built Environment* (originally published in 1996 by Oxford University Press).

## Disclosure statement

No potential conflict of interest was reported by the authors.

## Funding

This work was supported by the Wellcome Trust [grant number GR079946/B/06/Z], [grant number GR079946/Z/06/Z]; Singapore Ministry of Education Academic Research Fund Tier 1 [grant number R-109-000-156-112]; Singapore Ministry of Education Academic Research Fund Tier 2 [grant number MOE 2015-T2-1].

## Notes

1. Employment, homeownership, marriage and fatherhood were dominant masculine values that reportedly constitute the ‘package deal’ of successful white, middle-class American manhood.

2. Refer to Graham and Yeoh (2013) for more information on the study.

3. Young children completed a drawing activity instead of a survey.

4. The term ‘father-carer’ is a shortened phrase to describe fathers who have reported themselves as the main caregivers of the left-behind children in their wife’s absence. It does not imply that fathers outside of this study do not perform carework or that they are the only caregivers. However, Indonesian and Filipino men are generally not expected to assume the primary caregiving role when their wives are present.

5. Only 7.3% of main carers were identified as fathers in non-migrant households. Thailand is excluded due to the small sample of Thai mother-migrant households.

6. This is a comparatively higher rate than Senaratna’s (2012) and Save the Children’s (2006) studies where only 12.2 and 25.9% of the Sri Lankan fathers were reported as primary carers in mother-migrant households respectively. It is however lower than Hugo and Ukwatta’s (2010) 2008 study where over 80% of Sri Lankan fathers with migrant spouses were identified as the main carer.

7. Pseudonyms are used to protect the respondents’ anonymity.

8. The interviewees’ ages are captured at the point of the survey conducted in 2008.

9. RMMFC is an acronym used to represent returned-mother-migrant-father-carer households interviewed; MMFC for mother-migrant-father-carer households.

10. Conversely, fewer mother-carers of migrant husbands worked outside the home (29.9% in Indonesia and 27% in the Philippines) thus reflecting the different societal pressures on women to focus more on nurturing roles in the domestic sphere (Robinson 2000; Osteria 2011).
